# Association of miR-21, miR-126 and miR-605 gene polymorphisms with ischemic stroke risk

**DOI:** 10.18632/oncotarget.21316

**Published:** 2017-09-28

**Authors:** Yang Xiang, Jing Guo, You-Fan Peng, Tan Tan, Hua-Tuo Huang, Hong-Cheng Luo, Ye-Sheng Wei

**Affiliations:** ^1^ Department of Clinical Laboratory, The Affiliated Hospital of Youjiang Medical University for Nationalities, Baise 533000, Guangxi, China; ^2^ Department of Dermatology, The Affiliated Hospital of Youjiang Medical University for Nationalities, Baise 533000, Guangxi, China

**Keywords:** ischemic stroke, microRNAs, gene, single nucleotide polymorphisms, association analysis

## Abstract

We investigated whether three common microRNA polymorphisms (miR-21T>C [rs1292037], miR-126G>A [rs4636297] and miR-605T>C [rs2043556]) were associated with ischemic stroke (IS) risk in a Chinese population. The study population comprised 592 ischemic stroke patients and 456 normal controls. The polymorphisms were measured using Snapshot SNP genotyping assays and confirmed by sequencing. Relative expressions of miR-21, miR-126 and miR-605 were measured by quantitative real-time PCR. We found that miR-126 gene rs4636297 polymorphism was associated with decreased ischemic stroke risk (GA vs. GG: AOR=0.64, adjust P=0.025; AA vs. GG: AOR=0.32, adjust P=0.007; dominant model: AOR=0.58, adjust P=0.004). MiR-21 gene rs1292037 and miR-605 gene rs2043556 polymorphisms were not associated with ischemic stroke risk. In addition, compared with normal controls, serum miR-126 level was significantly decreased in ischemic stroke patients, while the miR-21 level was significantly increased. Importantly, patients carrying rs4636297 GA/AA genotypes had higher serum miR-126 level (P<0.05). MiR-126 gene rs4636297 polymorphism and serum miR-126/-21 levels are associated with ischemic stroke risk. Our data indicates that miR-126 and miR-21 play roles in the development of ischemic stroke.

## INTRODUCTION

Ischemic stroke is a common cerebral blood circulation disorder disease which is a serious threat to human health. It is the second leading cause of death in China [1]. Its growing trend of prevalence deserves the attention of medical community worldwide. Common risk factors, such as age, gender, smoking, hypertension and abnormal lipid metabolism account for part of the prevalence of ischemic stroke [2–5]. Genetic factors have been defined as important risk contributors to the pathogenesis of ischemic stroke [6–8].

MicroRNAs (miRNAs) are class of small non-coding single-stranded RNAs molecules of about 21-23 nucleotides in length, which involve in regulating gene expressions and contribute to various disease pathogenesis, such as inflammation, cancer, atherosclerosis, and ischemic stroke [9–13]. Atherosclerosis start from the dysfunction endothelial cells (ECs) which was regard to be a major cause of ischemic stroke. ECs enriched microRNAs, such as miR-21 and miR-126 have been found to be involved in the progression of atherosclerosis by modulating the function of ECs [14, 15]. For instance, miR-21 involved in the regulation of angiogenesis mediated by ECs [16]; microRNA-126-5p promotes endothelial proliferation and limits atherosclerosis by suppressing DIk1 [17]. In addition, the serum level of miR-21 and miR-126 were significantly changed in ischemic stroke and atherosclerosis patients [18–20]. The exact mechanism of the abnormal miR-21 and miR-126 expression remain unclear and genetic factors may play key roles in this process [21].

Single nucleotide polymorphisms in microRNAs were related to individual's susceptibility to a number of human diseases [22–24]. Polymorphisms in microRNAs were reported to be associated with ischemic stroke risk [25–27]. Recently, three common microRNA polymorphisms (miR-126G>A: rs4636297, chromosome 9, 139565150; miR-21T>C: rs1292037, chromosome 17, 57918908; miR-605T>C: rs2043556, chromosome 10, 53059406) have been investigated in a variety of human diseases [28–34]. Up to now, no report was performed to examine the association between these three common SNPs (i.e. rs4636297, rs2043556 and rs1292037) and ischemic stroke risk. Based on this background, we hypothesized that these three polymorphisms may relate to ischemic stroke risk. Therefore, we performed the study in a Chinese population, for the first time, to evaluate the association between rs4636297G>A, rs2043556T>C and rs1292037T>C polymorphisms and ischemic stroke susceptibility and to assess the associations between these three polymorphisms and the expression levels of miR-21/-126/-605.

## RESULTS

### Clinical characteristics of the study participants

The clinical characteristics of the study subjects were shown in Table 1. There is no significant difference between cases and controls with regard to age (P=0.884), gender (P=0.609) and HDL-C levels (P=0.200). Ischemic stroke patients were significantly more likely to smoke and have higher percentage of diabetes mellitus, hypertension, as well as high serum TC, TG and LDL-C levels (both P<0.05).

**Table 1:** Clinical characteristics of the study population

Variable	Stroke patients, n =592(%)	Control subjects, n =456(%)	*P* value
Age (mean ± SD)	64.7±11.5	64.0±13.5	0.884
Gender (M/F)	367/225	275/181	0.609
Diabetes mellitus	154(17.6)	55(12.1)	0.015
Hypertension	304(51.4)	155(34.0)	<0.001
Smoke	226(38.2)	142(26.8)	<0.001
Hyperlipidemia	290/302	128/328	<0.001
TC (mmol/L)	5.13±0.79	3.87±0.72	<0.001
TG (mmol/L)	1.85±0.89	1.48±0.52	<0.001
LDL-C(mmol/L)	2.63±0.99	2.16±0.97	<0.001
HDL-C(mmol/L)	1.62±0.48	1.66±0.51	0.200

Hyperlipidemia: Hyperlipidemia was defined as a high fasting serum total cholesterol level (> 6.22 mmol/L) or an antihyperlipidemic agent treatment history, TC: total cholesterol, TG: triglyceride, HDL-C: high-density lipoprotein cholesterol, LDL-C: low-density lipoprotein cholesterol.

### Genotype distributions of the three microRNAs polymorphisms in patients and controls

All of the polymorphisms have three genotypes respectively. The genotype and allele frequencies of rs4636297, rs1292037 and rs2043556 polymorphisms in case and control groups were shown in Table 2. The genotype distributions of the three polymorphisms in both cases and controls were in HWE (both P>0.05). The power of rs4636297polymorphism was 0.99. We calculated the adjusted odds ratio (AOR) from logistic regression analyses with respect to age, gender, hypertension, diabetes mellitus, smoking, TC, TG, LDL-C and HDL-C. Compared with rs4636297 GG genotype, rs4636297 GA and AA genotypes were significantly associated with decreased risk of ischemic stroke (GA vs. GG: AOR=0.64, 95% CI, 0.44-0.95, P=0.025; AA vs. GG: AOR=0.32, 95% CI, 0.14-0.74, P=0.007; dominant model, AA+GA vs. GG: AOR=0.58, 95% CI, 0.40-0.84, P=0.004). However, rs1292037 and rs2043556 polymorphisms were not significantly associated with ischemic stroke risk.

**Table 2:** Genotype frequencies of microRNAs polymorphisms between ischemic stroke patients and control subjects

Polymorphisms	Stroke, n =592	Control, n=456	OR(95% CI)	P	AOR^*^ (95% CI)	P^*^
**miR-126 rs4636297G/A**						
GG	430(72.6)	275(60.3)	1.000(reference)		1.000(reference)	
GA	147(24.8)	154(33.8)	0.61(0.47-0.80)	<0.001	0.64(0.44-0.95)	0.025
AA	15(2.5)	27(5.9)	0.36(0.19-0.68)	0.001	0.32(0.14-0.74)	0.007
Dominant model(AA+GA vs. GG)		0.57(0.44-0.74)	<0.001	0.58(0.40-0.84)	0.004	
Recessive model(AA vs. GA+GG)		0.41(0.22-0.79)	0.006	0.37(0.16-0.82)	0.015	
Allele A frequency	177(14.9)	208(22.8)				
**miR-21 rs1292037T/C**						
TT	172(29.1)	152(33.3)	1.000(reference)		1.000(reference)	
CT	304(51.4)	229(50.2)	1.12(0.89-1.55)	0.259	1.21(0.81-1.80)	0.349
CC	116(19.6)	75(16.4)	1.37(0.95-1.97)	0.091	1.47(0.86-2.50)	0.159
Dominant model(CC+CT vs. TT)			1.22(0.94-1.59)	0.137	1.28(0.88-1.86)	0.204
Recessive model(CC vs. CT+TT)			1.24(0.90-1.71)	0.191	1.37(0.85-2.21)	0.192
Allele C frequency	536(45.3)	379(41.6)				
**miR-605 rs2043556 T/C**						
TT	332(56.1)	276(60.5)	1.000(reference)		1.000(reference)	
CT	232(39.2)	153(33.6)	1.26(0.97-1.63)	0.080	1.41(0.98-2.04)	0.093
CC	28(4.7)	27(5.9)	0.86(0.50-1.50)	0.598	0.65(0.29-1.50)	0.316
Dominant model(CC+CT vs. TT)			1.20(0.94-1.54)	0.148	1.31(0.92-1.86)	0.140
Recessive model(CC vs. CT+TT)			0.79(0.46-1.36)	0.385	0.60(0.27-1.34)	0.212
Allele C frequency	288(24.3)	207(22.7)				

OR, odds ratio; AOR, adjusted odds ratio; 95% CI, 95% confidence interval

^*^The AOR on the basis of risk factors such as age, gender, hypertension, diabetes mellitus, TC, TG, LDL-C, HDL-C and smoking.

### Stratified analyses

Stratified analyses based on age, gender, hypertension, smoke, diabetes mellitus, and hyperlipidemia were performed to find additional clinical significance. According to the results, we failed to find any association between these three polymorphisms and clinical characteristics of ischemic stroke. (Table 3)

**Table 3:** Stratified analysis of miR-126, miR-21, and miR-605 polymorphisms on ischemic stroke Risk

Variables	miR-126 (GA+AA) vs. GG		miR-21(TC+CC) vs.TT		miR-605 (TC+CC) vs.TT	
	AOR^*^	P^*^	AOR^*^	P^*^	AOR^*^	P^*^
Age						
<60	1.43 (0.81-1.70)	0.210	0.87(0.55-1.39)	0.560	1.04(0.67-1.61)	0.858
≥60	1.14(0.78-1.94)	0.180	1.40(0.98-1.98)	0.063	1.23(0.88-1.72)	0.218
Gender						
Male	1.37(0.84-1.82)	0.210	1.33(0.93-1.91)	0.117	1.14(0.81-1.61)	0.446
Female	1.10(0.61-1.58)	0.249	1.02(0.65-1.59)	0.945	1.23(0.81-1.87)	0.326
Diabetes mellitus						
Yes	0.53(0.27-1.74)	0.165	0.97(0.48-1.96)	0.934	1.09(0.57-2.07)	0.803
No	1.22(0.76-1.84)	0.178	1.23(0.91-1.68)	0.181	1.71(0.88-1.56)	0.284
Hypertension						
Yes	1.11(0.65-1.88)	0.710	1.34(0.87-2.06)	0.189	1.22(0.80-1.84)	0.356
No	1.22(0.73-1.90)	0.211	1.17(0.86-1.59)	0.327	0.85(0.58-1.25)	0.406
Smoke						
Yes	1.57(0.93-2.66)	0.193	1.51 (0.92-2.47)	0.102	1.10(0.68-1.77)	0.697
No	1.38(0.91-1.81)	0.231	1.05 (0.75-1.46)	0.797	1.22(0.89-1.68)	0.221
Hyperlipidemia						
Yes	0.85(0.54-1.34)	0.476	0.84(0.54-1.32)	0.454	0.82(0.52-1.28)	0.380
No	1.16(0.82-1.63)	0.396	1.18(0.53-1.8)	0.335	1.78(0.84-1.65)	0.349

AOR, adjusted odds ratio; 95% CI, 95% confidence interval

^*^The AOR on the basis of risk factors such as age, gender, hypertension, diabetes mellitus, hyperlipidemia and smoking.

### Relative expressions of miR-21, miR-126 and miR-605

**Figure 1:**
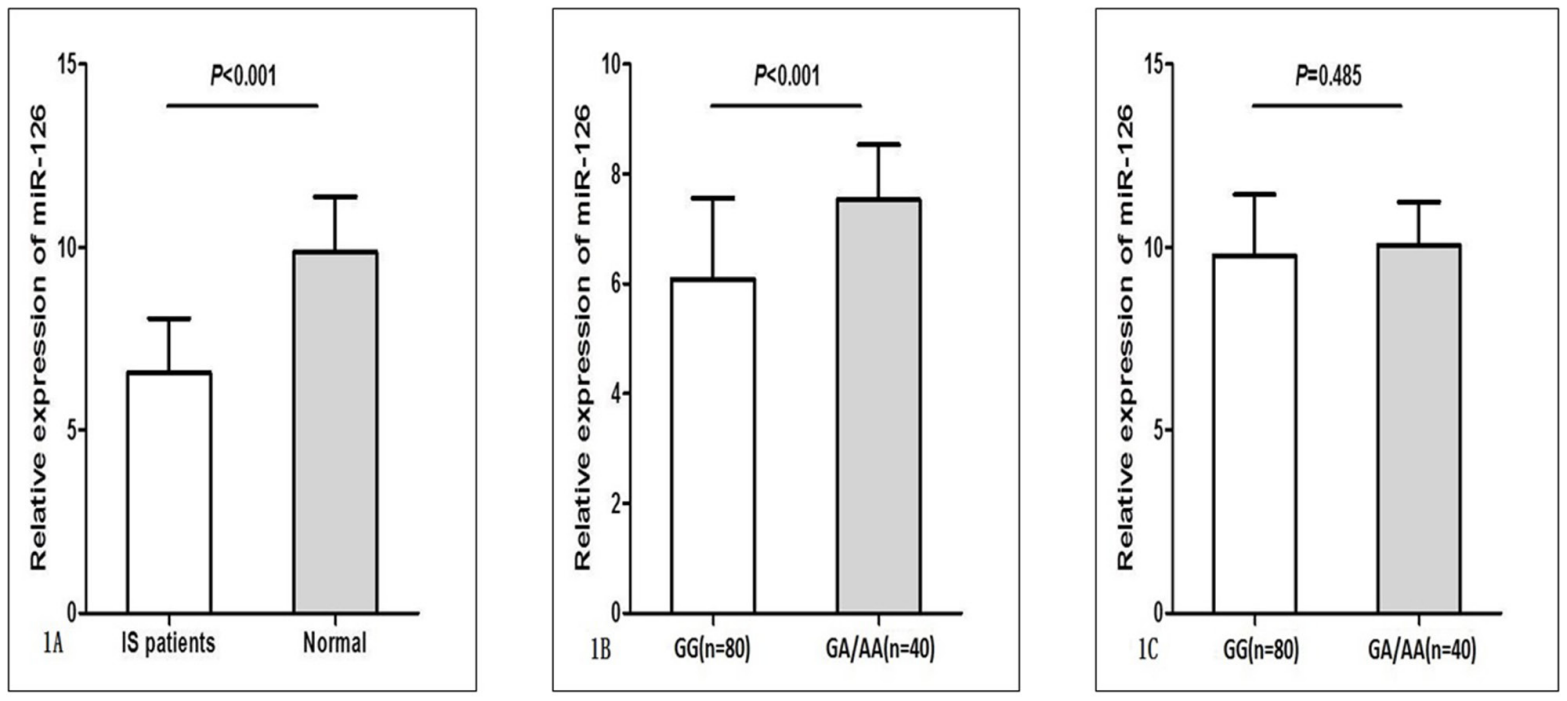
Relative expression of miR-126 (normalized to U6) in ischemic stroke (IS) patients (n = 120) and normal controls (n = 120) **(A)** Serum levels of miR-126 were significantly decreased in IS patients compared with normal controls. **(B)** IS patients carrying rs4636297GA/AA genotypes had significant high serum levels of miR-126. **(C)** In normal controls rs4636297G>A polymorphism did not associated with serum miR-126 level. Data were presented as mean ± standard error.

Relative serum miR-21, miR-126 and miR-605 levels were detected in both ischemic stroke patients and control subjects. Serum levels of miR-126 were significantly decreased in stroke patients compared with normal controls (IS vs. control=6.57±1.50 vs. 9.86±1.92, P<0.001; Figure 1A). Patients carrying rs4636297GA/AA genotypes had significant higher serum levels of miR-126 (GG vs. GA/AA=6.08±1.47 vs. 7.53±2.00, P<0.001; Figure 1B). In normal controls rs4636297G>A polymorphism did not associated with serum miR-126 level (GG vs. GA/AA=9.77±1.67 vs. 10.6±1.19, P = 0.485; Figure 1C). MiR-21 was significantly increased in ischemic stroke patients (IS vs. control=34.65±7.26 vs. 26.89±6.81, P<0.001). But, rs1292037T>C polymorphism did not associated with the expression of miR-21(IS: TT vs. TC/CC=32.5±5.80 vs. 35.27±8.70, P=0.203; Control: TT vs. TC/CC=25.64±6.94 vs. 27.51±6.75, P=0.320, respectively). Serum miR-605 levels between patients and controls showed no significant difference (IS vs. control=21.03±8.53 vs. 19.21±9.15, P=0.262). Besides, serum miR-605 levels did not associated with any genotype of rs2043556 T>C polymorphism (IS: TT vs. TC/CC=21.26±9.19 vs. 20.70±7.68, P=0.802; Control: TT vs. TC/CC=20.12±8.65 vs. 17.93±9.85, P=0.363, respectively).

## DISCUSSION

This is the first study to evaluate the association between three common microRNA polymorphisms (rs4636297G>A, rs1292037T>C and rs2043556T>C) and ischemic stroke risk in a Chinese population. We demonstrated for the first time that miR-126 gene rs4636297G>A polymorphism was associated with a reduced risk of ischemic stroke. Moreover, patients carrying rs4636297 GA/AA genotypes had a higher level of miR-126. These results indicated that miR-126 gene rs4636297G>A polymorphism may play a role in the progression of ischemic stroke.

Atherosclerosis is a hyperlipidemia-induced chronic inflammatory process of the arterial wall, resulting from the disturbed endothelial cells (ECs). It was regard to be a major cause of stroke. The integrity of the endothelial monolayer is fundamental for the homoeostasis of the vascular system, and functional endothelial cells are also required for the growth of new blood vessels during neovascularization [16], endothelial cell functions and angiogenesis are critically regulated by microRNAs, including miR-126, miR-143 and miR-145 et al [35]. The endothelial cell-specific miR-126 is transferred in micro-vesicles from apoptotic endothelial cells and plays important roles in vascular inflammation related diseases, including chronic kidney disease [36, 37], atherosclerosis [38, 39] and stroke [10]. In ischemic stroke etiology, miR-126 modulates the pathogenic processes of atherosclerosis thereby affecting the stroke progression [40]. These studies indicated that miR-126 may play important role in the development of ischemic stroke. However, the association between rs4636297G>A polymorphism in miR-126 gene and ischemic stroke risk remains unclear.

MiR-126 gene is located in chromosome 9q34.3 within the host gene encoding epidermal growth factor-like protein 7 (EGFL7) [41]. Several reports have explored the association between rs4636297G>A polymorphism and human disease susceptibility. Results were inconsistent. Rs4636297 G>A polymorphism was not associated with breast cancer and non-small cell lung cancer risk [29, 30]. While, another report indicated that A allele of rs4636297G>A can impair the inhibition of mature miR-126 to increase VEGF promotion and increase the risk for developing sight threatening diabetic retinopathy (STDR) [34]. In addition, a functional study revealed that both major G and minor A allele of rs4636297G>A can blocks the processing of primary miR-126 to precursory miR-126 and resulting in significantly reduced mature miR-126 expression, although the block effect of minor A allele is less effective [21]. In this study, we investigated the association between miR-126 gene rs4636297G> A polymorphism and ischemic stroke risk and the miR-126 expression. According to the results, we found that miR-126 gene rs4636297G>A polymorphism was associated with a reduced risk of ischemic stroke and serum level of miR-126 was significantly reduced in stroke patients. Besides, patients carrying GA/AA genotypes had higher miR-126 levels compared with those carrying GG genotypes. With regard to the potential mechanism, we speculated that rs4636297G>A polymorphism may exert influence on the processing of primary miR-126 to precursory miR-126 in ischemic stroke patients. Owing to the relative low inhibiting functions of A allele, patients carrying GA/AA genotypes had a higher level of miR-126 compared with those carrying GG genotype. However, we should not ignore the fact that in normal controls the miR-126 levels did not associated with any rs4636297G>A genotypes. The reason of this difference remains unclear; a possible explanation may be that the miR-126 expression is inducible and its expression is changed after stimulation. The miR-126 levels may be determined by both genetic and inducing factors. Further gene-environment interaction analysis may better reveal the role of miR-126 gene polymorphisms in the etiology of ischemic stroke.

Vascular smooth muscle cell (VSMC)-enriched miR-21 was involved in the pathogenesis of atherosclerosis related disease [42]. Consistent with previous studies, we also detected significantly increased serum miR-21 levels in ischemic stroke patients. However, the current data did not support the association between miR-21 gene rs1292037 T>C polymorphism and ischemic stroke risk.

Rs2043556T/C polymorphism of miR-605 gene was related to certain human diseases [31, 32]. A recent report showed that rs2043556T/C did not associated with the recurrence of ischemic stroke [43]. In present study we found that rs2043556T/C also did not associated with the ischemic stroke risk. Serums miR-605 levels also showed no significant difference between patients and controls. Besides, rs2043556T/C polymorphism did not associated with serum miR-605 level. These findings suggested that miR-605 gene rs2043556T/C polymorphism may not play role in the progression of ischemic stroke.

Although our results are promising, it should be noted that our study have several limitations. First, all subjects may not give the same battery of diagnostic clinical and laboratory tests; therefore, potential selection bias could not be ruled out and might influence the interpretation of the results. Further independent validation studies are required to confirm the stability of the current findings. Second, relative small sample size was selected to detect the serum microRNAs levels; the results should be further validated in larger sample studies. Third, for the loss of stroke subtype information, we could not analyze the role of microRNA polymorphisms in different subtypes of ischemic stroke. Finally, more advanced statistical methods and validation experiments should be applied to subsequent studies to explore the exact regulatory mechanism of miRNA gene polymorphisms in ischemic stroke.

In summary, miR-126 gene rs4636297G>A polymorphism was associated with decreased risk of ischemic stroke. Significant changes in the circulating levels of miR-126 and miR-21 in ischemic stroke patients indicating that miR-126 and miR-21 might be potential diagnosis biomarkers or therapeutic targets for ischemic stroke.

## MATERIALS AND METHODS

### Study subjects

The study was performed with the approval of the ethics committee of the Affiliated Hospital of Youjiang Medical University for Nationalities, and written informed consent was obtained from all the subjects. The experiments were performed in accordance with relevant guidelines and regulations. The study included 592 ischemic stroke patients (367 males and 225 females) from the Department of Neurology, Affiliated Hospital of Youjiang Medical University for Nationalities, Guangxi, China between January 2013 and September 2015. The diagnosis of ischemic stroke was based on the appearance of a new and abrupt focal neurological deficit, with neurological symptoms and signs persisting for more than 24h. Ischemic stroke was confirmed by the positive findings by head CT or MRI according to the International Classification of Disease (9th Revision, codes 430 to 438). Patients with hemorrhagic stroke, coronary heart disease, autoimmune diseases, cancers or other serious diseases were excluded from the study. The 456 sexes- and age- matched controls (275 males and 181 females) were selected from volunteers who underwent physical examination in health examination center, Affiliated Hospital of Youjiang Medical University for Nationalities, Guangxi, China during the same period. Confirmed by head CT, MRI scanning (negative imaging finding) and thorough clinical and laboratory evaluation, the healthy volunteers meeting the same exclusion criteria as the cases were enrolled in this study.

Clinical information, such as hypertension, diabetes, fasting serum levels of total cholesterol (TCH), triglyceride (TG), high-density lipoprotein cholesterol (HDL-C), and low-density lipoprotein cholesterol (LDL-C) was abstracted from medical record review. All study subjects were Chinese and resided in the same geographic area in Guangxi China.

### SNPs selection

We selected three common microRNA polymorphisms (miR-21T>C [rs1292037], miR-126G>A [rs4636297] and miR-605T>C [rs2043556]) which have been identified in a variety of human diseases [28–34].

### DNA extraction

Genomic DNA was extracted from blood leucocytes by using a whole-blood genome DNA extraction reagent kit [Axygene Biotechnology (Hangzhou) Limited (Hangzhou City, China)], following the manufacturers’ instructions.

### Determination of genotype

The method of Snapshot SNP genotyping assay was taken to detect the allele and genotype frequencies. To confirm the genotyping results, PCR-amplified DNA samples were examined by DNA sequencing and the results were 100% concordant. The PCR primers were designed based on the GenBank reference sequence (accession no. miR-126: NC_000009.12; miR-21: NC_000017.11; miR-605: NC_000010.11).

### Quantitative PCR of microRNAs

In all patients, plasma samples were collected within 12h after symptom onset. After an overnight fasting, venous blood samples of control population were collected. Plasma was isolated by centrifugation and was maintained at -70 °C until use. 120 stroke patients and 120 normal controls were randomly selected to detecting serum microRNAs levels. Total RNA was isolated using a commercial kit (Qiagen, Hilden, Germany) following the manufacturer's protocol. One microgram of total RNA was reverse transcribed into cDNA utilizing reverse transcription kits from Ribobio Corp. (Guangzhou, China). After cDNA conversion, quantitative PCR was done using Power SYBR Master Mix and ABI 7500 real-time PCR machine (Applied Bio-systems, CA, and USA). We purchased the primers from Ribobio Corp., (Guangzhou, China). U6 was used as an internal control. Relative expression levels of microRNAs were computed using comparative Ct method (2^−ΔCt^).

### Statistical analysis

The SPSS statistical software package version 17.0 was used for the statistical analysis. Continuous variables were displayed as mean ± SD. If the data were normally distributed, the Student's t-test was used; otherwise, Mann-Whitney U test was used. Categorical variables were expressed as proportions and compared by using chi-squared test. Hardy–Weinberg equilibrium (HWE) was tested by chi-squared test. The association of the miR-126G>A, miR-21T>C and miR-605T>C polymorphisms and risk of ischemic stroke was evaluated by odds ratios (ORs) with 95% confidence interval (CIs). For multivariate analyses, logistic regression analyses were used to adjust for possible confounders, including age, sex, hypertension, diabetes mellitus, smoking, TCH, TG, HDL-C, and LDL-C. The power analysis was performed using NCSS-PASS 11.0. Statistical significance was accepted at the P<0.05 level.
